# Quality Assessment of Mixed and Ceramic Recycled Aggregates from Construction and Demolition Wastes in the Concrete Manufacture According to the Spanish Standard ^†^

**DOI:** 10.3390/ma7085843

**Published:** 2014-08-13

**Authors:** Desirée Rodríguez-Robles, Julia García-González, Andrés Juan-Valdés, Julia Mª Morán-del Pozo, Manuel I Guerra-Romero

**Affiliations:** Superior and Technical School of Agricultural Engineering, University of Leon, Avda. Portugal 41, 24071 Leon, Spain; E-Mails: julia.garcia@unileon.es (J.G.-.G.); andres.juan@unileon.es (A.J.-V.); julia.moran@unileon.es (J.M.M.P.); ignacio.guerra@unileon.es (M.I.G.-R.)

**Keywords:** characterization, composition, construction and demolition waste (CDW), ceramic aggregate, mixed aggregate, recycled concrete

## Abstract

Construction and demolition waste (CDW) constitutes an increasingly significant problem in society due to the volume generated, rendering sustainable management and disposal problematic. The aim of this study is to identify a possible reuse option in the concrete manufacturing for recycled aggregates with a significant ceramic content: mixed recycled aggregates (MixRA) and ceramic recycled aggregates (CerRA). In order to do so, several tests are conducted in accordance with the Spanish Code on Structural Concrete (EHE-08) to determine the composition in weight and physic-mechanical characteristics (particle size distributions, fine content, sand equivalent, density, water absorption, flakiness index, and resistance to fragmentation) of the samples for the partial inclusion of the recycled aggregates in concrete mixes. The results of these tests clearly support the hypothesis that this type of material may be suitable for such partial replacements if simple pretreatment is carried out. Furthermore, this measure of reuse is in line with European, national, and regional policies on sustainable development, and presents a solution to the environmental problem caused by the generation of CDW.

## 1. Introduction

Although the construction sector’s important contribution to the development of society is widely acknowledged, the construction industry is also perceived as a major cause of environmental degradation. Special concern rests in the European construction industry, as, besides being one of the largest consumers of natural resources—more than 50% of European natural resources [1], it is also a major contributor in the waste scenario—about 33% of the waste generated annually [2], as large quantities of the raw minerals employed end up in landfills. Consequently, the construction sector cannot remain aloof to the widespread social demand for greater respect for the environment, and must assume the task of reducing the impact caused by building work.

Construction and demolition waste (CDW) constitutes an increasingly significant problem in society, not so much because of its hazardous nature, as it can be inert, but because of the volume generated, which renders sustainable management and disposal problematic. However, this waste has a very high potential for recovery, although Spain (30%) is still situated behind other European countries in terms of sustainable CDW management [3].

Research on the use of recycled materials has become a widespread tendency. Numerous studies have investigated the possibilities of using recycled aggregates in concrete mixes as partial replacement of the conventional coarse aggregate (gravel), although the majority of them have basically focused on the use of aggregates recovered from concrete (RCA) [4,5,6,7,8,9,10], as their properties do not differ as much of the natural aggregates, and their use is supported by most of the standards which allow the use of secondary materials in the concrete manufacture, *i.e.*, in Spain, the current legislation—the Spanish Code on Structural Concrete (EHE-08) [11] only allows this type of recycle aggregate for its inclusion up to 20% in concrete mixes, excluding the mixed recycled aggregates (MixRA), even for non-structural concretes. However, recycled aggregates obtained from crushed concrete, which despite having received most attention, only account for 15% of the CDW generated, and 30% of the CDW marketed [3].

CDW includes a wide range of inert materials, as their composition is affected by numerous factors, including the raw materials and construction products used, the architectural techniques, and the local construction and demolition practices. Given the typical composition of CDW, there are basically two types of recycled aggregates: which are obtained from crushed concrete (RCA) and those comprising mixed components with varying percentages of ceramic material (MixRA and ceramic recycled aggregates (CerRA)). In Spain, as in other Mediterranean countries, such as Portugal, Italy, or Greece, the construction of buildings is usually based on ceramic elements combined with mortar and concrete [12]. As determined in the Spanish National Plan of Construction and Demolition Waste 2001–2006 [13], one of the most significant fractions of the CDW produced is that it is composed of stone materials (bricks, roof tiles, ceramic materials and products, slabs, concrete, *etc.*), of which, more than half (54%) corresponds to the ceramic fraction, indicating the importance of treatment and recovery of this kind of waste. Furthermore, recycled aggregates also include small amounts of other materials (impurities) depending on the origin of the waste and the treatment process carried out in the recycling plant.

In this research work, as an attempt to identify a possible means of reusing this CDW, several samples of recycled aggregates with different ceramic contents were tested to assess their suitability for inclusion in concrete mixes as a partial replacement of the conventional coarse aggregate. In order to do so, the traditional method of characterizing recycled CDW aggregate was employed. This characterization was based on determining the macroscopic composition in terms of weight of the different components in the samples and measuring the key physical properties of the recycled aggregates (particle size distributions, fine content, sand equivalent, density, water absorption, flakiness index, and resistance to fragmentation).

## 2. Materials

**Figure 1:**
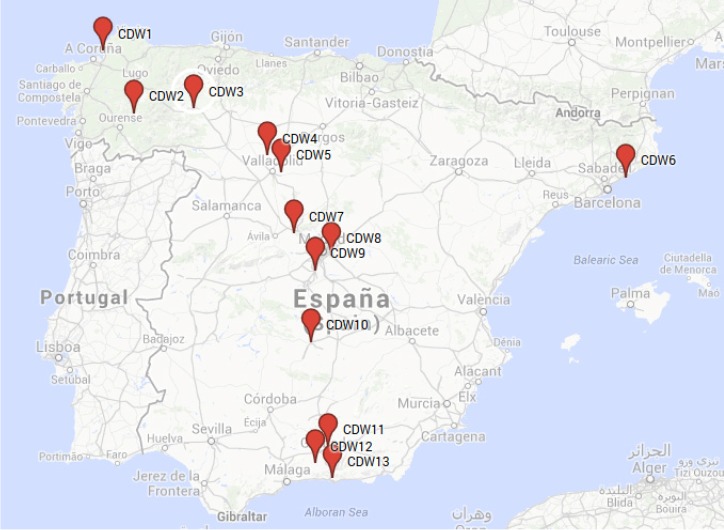
Spatial distribution of the recycled aggregates samples. Adapted from Google Maps [14].

In this study, thirteen samples of recycled aggregates from CDW were analyzed. The samples were collected from ten CDW treatment plants located in different Spanish provinces (Figure 1).

## 3. Methods

The characteristics of the aggregates used in the manufacture of concrete largely determine the properties of the final product. Thus, in order to get quality recycled concrete, it is necessary that the recycled aggregates used meet the requirements of the standard UNE-EN 12620:2003 + A1: 2009 [15] and the Chapter VI: Materials of the EHE-08 [11].

In order to perform the characterization of the recycled aggregates, a representative quantity of all the samples was selected, following the specifications in UNE-EN 932-1:1997 [16], and was subjected to the following tests: composition content (UNE-EN 933-11:2009/AC:2010 [17]), particle size (EHE-08 [11] and UNE-EN 933-1:2012 [18]), quantity of fines (EHE-08 [11]), sand equivalent (UNE-EN 933-8:2012 [19]), flakiness index (UNE-EN 933-3:2012 [20]), density and water absorption (UNE-EN 1097-6:2001/A1:2006 [21]) and Los Angeles test (UNE-EN 1097-2:2010 [22]).

Finally, by comparing the results obtained and the guidelines established by the aforementioned Spanish standards, it was possible to assess the suitability of the materials for use as recycled aggregate in concrete manufacturing.

## 4. Results and Discussion

### 4.1. Composition

The constituents found in the recycled coarse aggregates (4/40 mm) are shown in Table 1.

**Table 1:** Composition of recycled aggregates (coarse fraction) from construction and demolition waste (CDW), based on UNE-EN 933-11 [17]. *R*c: concrete, mortar and natural aggregates with mortar attached; *R*u: unbound natural aggregates without mortar attached; *R*b: ceramics (brick, tiles…); *R*g: glass; *R*a: asphalt; *X*_1_: gypsum; and *X*_2_: other impurities (wood, plastic, metals).

Sample ^a^	*R*c	*R*u	*R*b	*R*g	*R*a	*X*_1_	*X*_2_
CDW1	49.14	29.47	16.51	0.17	4.38	0.09	0.25
CDW2	28.67	33.91	33.56	0.11	0.83	2.64	0.28
CDW3	33.07	37.65	28.43	0.04	0.29	0.22	0.31
CDW4	46.69	20.02	31.41	0.08	1.33	0.34	0.14
CDW5	56.41	18.08	23.96	0.00	1.18	0.21	0.16
CDW6	14.03	45.44	38.08	0.00	1.35	0.65	0.45
CDW7	44.11	17.51	33.56	0.75	0.44	3.48	0.16
CDW8	32.01	42.69	21.42	0.58	1.02	1.84	0.44
CDW10	36.35	9.33	49.89	0.01	0.17	4.12	0.13
CDW11	12.76	21.38	64.75	0.00	0.86	0.12	0.14
CDW12	46.86	23.83	24.08	0.00	1.05	3.81	0.35
CDW13	34.62	38.72	21.91	0.00	3.10	1.55	0.10
Mean	36.23	28.17	32.30	0.14	1.33	1.59	0.24
Standard deviation	13.45	11.47	13.58	0.25	1.22	1.56	0.12
Maximum	56.41	45.44	64.75	0.75	4.38	4.12	0.45
Minimum	12.76	9.33	16.51	0.00	0.17	0.09	0.10

^a^ Results of Sample CDW9 are not available.

The recycled aggregates were composed of the following materials: concrete and mortar, natural aggregates, ceramics, asphalt, glass, gypsum, and impurities, such as wood, plastic, and metal. The data showed that the predominant material was concrete, with mean values of 36.23%, followed by materials of a ceramic nature, which constituted 32.30% on average, and the unbound aggregates with a mean value of 28.17%.

**Figure 2:**
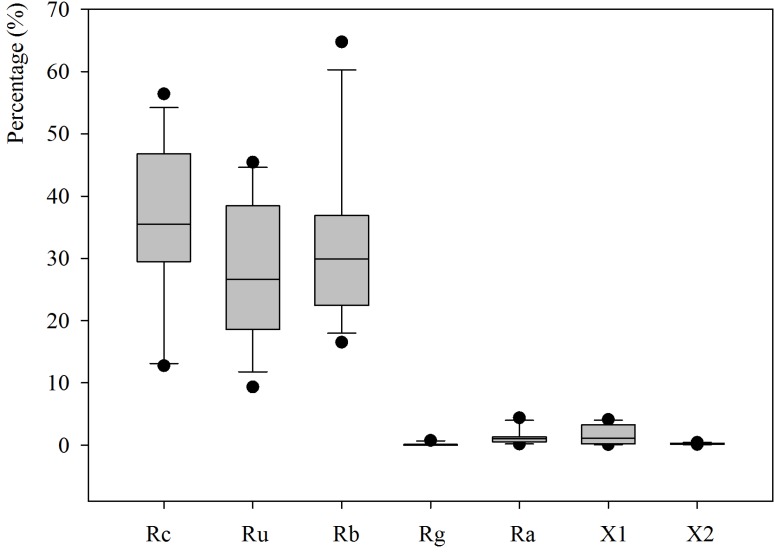
Boxplots showing the different components of the recycled aggregates.

However, as it can be observed in Table 1 and Figure 2, there is a wide variation in the composition between the different samples. The percentage of concrete content (natural aggregate and attached mortar) ranged between 12.76% and 56.41%, the natural aggregates without cement attached ranged between 9.33% and 45.44%, and ceramic particles content ranged between 16.51% and 64.75%. Glass content was lower than 1% for all the recycled aggregates. Asphalt content was lower than 4.50% for all samples, thus, no significant problems should be expected, since some studies [23,24] indicate that decrease of the compressive strength is due to a higher content of asphalt. In terms of gypsum content, the values ranged between 0.09% and 4.12%, which could generate some problems, specifically in Samples CDW2, CDW10, and CDW12, since, in a study conducted by Agrela *et al.* [9], it was found that recycled aggregates with a gypsum content, higher than 1.67%, should be rejected due to the lack of compliance with the 0.8% content limit for soluble sulphates in acid established in the Spanish and European specifications.

Lastly, some of the recycled aggregates (CDW2, CDW6, CDW7, CDW8, CDW10, CDW12, and CDW13) contained more than 1% of impurities (*X*_1_ + *X*_2_), thus, a pretreatment to remove the mentioned contaminations is necessary in order to comply with the EHE-08 requirements.

This determination of the component proportions in the recycled coarse aggregates allowed a classification based on its ceramic content. Two groups of CDW treatment plants were established according to the following limits of ceramic particles contents in weight:

- MixRA: samples with ceramic content inferior to 30%. Six facilities produce this type of recycled aggregate (CDW1, CDW3, CDW5, CDW8, CDW12, and CDW13).
- CerRA: samples with ceramic content superior to 30%. The rest of the CDW treatment plants produce this type of recycled aggregate (CDW2, CDW4, CDW6, CDW7, CDW10, and CDW11).

### 4.2. Physical-Mechanical Properties

**Figure 3:**
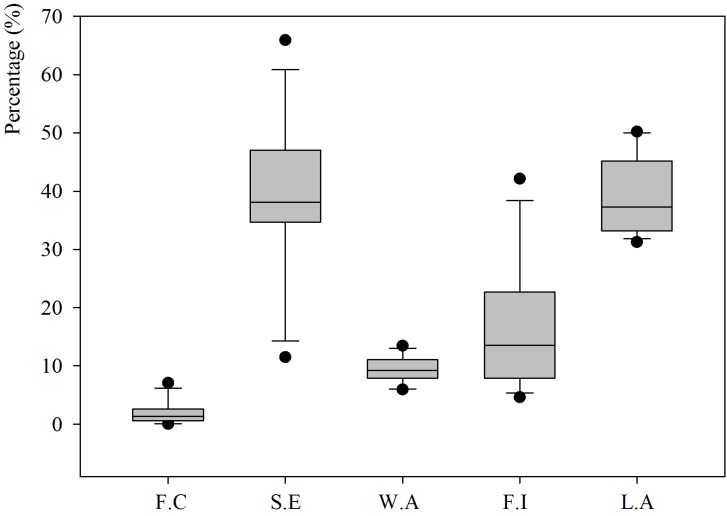
Boxplots showing the fine content (*F.C*), sand equivalent (*S.E*), water absorption (*W.A*), flakiness index (*F.I*), and Los Angeles test value (*L.A*) of the recycled aggregates.

**Figure 4:**
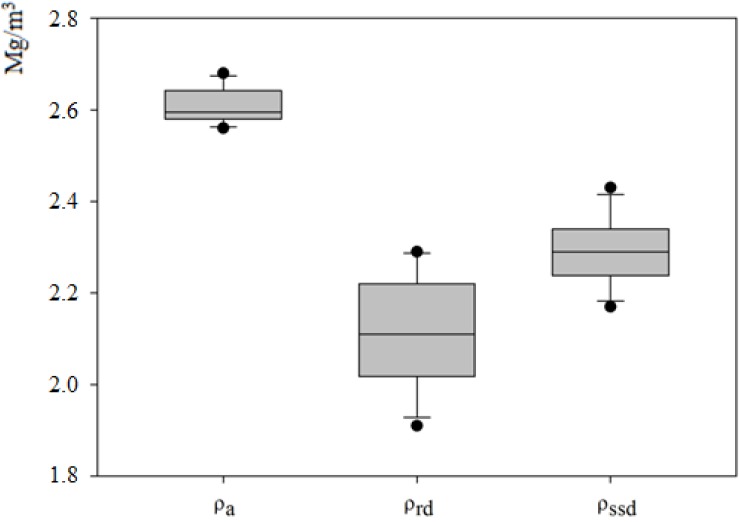
Boxplots showing the apparent density (ρ_a_), after oven-drying density (ρ_rd_), and saturated surface density (ρ_ssd_) of the recycled aggregates.

However, the previous study of composition and classification of the recycled aggregates did not determine the suitability of the material for its use in the manufacturing of concrete, being that the technical quality of the material is what determines its suitability. Therefore, Table 2 shows the results obtained for the tests conducted, and Figure 3 and Figure 4 illustrate the margin in which the results fluctuate.

**Table 2:** Results of the physical-mechanical test conducted according to the EHE-08 [11]. *D*: maximum particle size; *d*: minimum particle size; *D*/*d*: particle size ratio; *F.C*: fine content; *S.E*: sand equivalent; ρ_a_: apparent density; ρ_rd_: after oven-drying density; ρ_ssd_: saturated surface density; *W.A*: water absorption; *F.I*: flakiness index; and *L.A*: Los Angeles test value.

Sample	*D* (mm)	*d* (mm)	*D*/*d*	*F.C* (%)	*S.E* (%)	ρ_a_ (Mg/m^3^)	ρ_rd_ (Mg/m^3^)	ρ_ssd_ (Mg/m^3^)	*W.A* (%)	*F.I* (%)	*L.A* (%)
CDW1	31.50	10.00	3.15	1.82	35.40	2.57	2.14	2.31	7.84	14.94	49.64
CDW2	31.50	0.40	78.75	2.84	20.80	2.60	2.28	2.28	9.40	29.57	46.61
CDW3	31.50	0.40	78.75	4.08	65.90	2.65	2.29	2.43	5.96	24.67	33.23
CDW4	31.50	0.32	100.00	1.28	48.30	2.56	2.07	2.26	9.11	12.25	31.27
CDW5	31.50	1.25	25.20	0.60	49.00	2.58	2.13	2.30	8.14	9.61	37.85
CDW6	12.50	0.06	198.40	7.09	38.80	2.58	1.91	2.17	13.46	4.64	38.47
CDW7	20.00	4.00	5.00	0.04	38.10	2.59	2.00	2.23	11.31	14.75	40.99
CDW8	31.50	2.00	15.80	0.78	45.30	2.64	2.32	2.44	5.21	11.98	35.54
CDW9	31.50	2.00	15.80	0.44	26.70	2.56	2.16	2.32	7.27	11.85	35.96
CDW10	31.50	16.00	1.97	0.28	43.27	2.58	1.97	2.21	12.09	16.67	50.20
CDW11	31.50	10.00	3.20	0.80	11.5	2.62	2.07	2.28	10.27	42.13	33.14
CDW12	12.50	4.00	3.10	1.42	38.10	2.66	2.16	2.35	8.65	7.07	33.21
CDW13	12.50	4.00	3.10	1.54	34.50	2.68	2.09	2.31	10.42	7.36	36.76
Mean	26.23	4.19	40.94	1.77	38.13	2.61	2.12	2.30	9.16	15.96	38.68
Standard deviation	8.44	4.88	58.58	1.95	13.64	0.04	0.12	0.08	2.36	10.48	6.38
Maximum	31.5	16	198.4	7.09	65.9	2.68	2.32	2.44	13.46	42.13	50.2
Minimum	12.5	0.06	1.97	0.04	11.5	2.56	1.91	2.17	5.21	4.64	31.27

#### 4.2.1. Particle Size Curves

**Figure 5:**
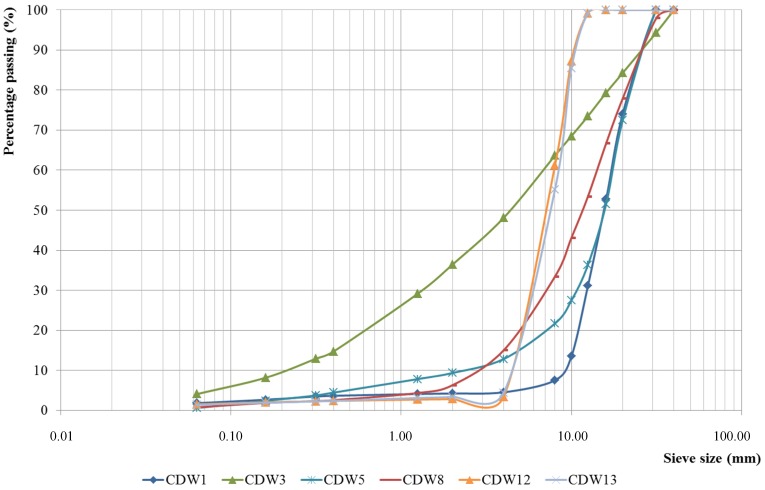
Particle size distribution curves of mixed recycled aggregates (MixRA).

**Figure 6:**
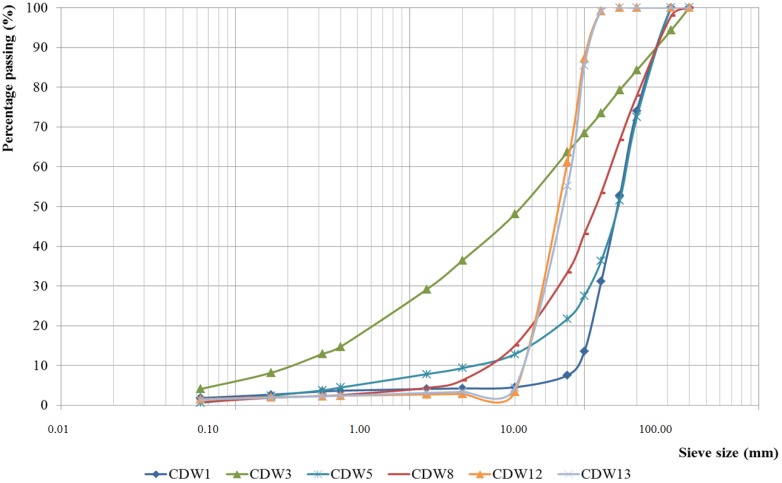
Particle size distribution curves of ceramic recycled aggregates (CerRA).

Particle size distributions of the MixRA and CerRA are shown in Figure 5 and Figure 6, respectively.

The size distribution curves of all materials are continuous and non-uniform, indicating that the granulometry of the aggregate is correct in all sizes, which allows a greater margin for interaction between the particles and produces a greater degree of compactness and mechanical strength in the concrete [25].

As indicated in the general requirements of the EHE-08 [11], Table 2 shows the maximum (*D*) and minimum (*d*) size of aggregates, and also depicts the relationship between both sizes. The *D*/*d* ratio is greater than 1.4, the limit stipulated in the EHE-08, for all the samples.

However, since the EHE-08 [11] defines coarse aggregate as particles with a minimum size of 4 mm, pretreatment of those samples, which do not meet this requirement, will be necessary (sieving to separate fines) before they can be employed as aggregate in concrete mixes.

#### 4.2.2. Fine Content

The EHE-08 [11] establishes the need to study the quantity of fines (*F.C*), placing an upper limit of 1.5% for coarse aggregates. This is an extremely important test, since a high amount of fines would prevent good paste adhesion and encourage concrete failure [26].

The results obtained, shown in Table 2, indicated that five of the samples (CDW1, CDW2, CDW3, CDW6, and CDW13) did not meet requirements. Nevertheless, the EHE-08 [11] allows their use under the restrictions imposed in Article 31.1 Furthermore, Etxeberria *et al.* [4] recommended that a maximum limit for recycled aggregate fines of between 2% and 5% should not be exceeded, and all the samples tested fell below this limit, with the exception of CDW6, which would need to be pre-treated through sieving to reduce the fine fraction in the sample.

#### 4.2.3. Sand Equivalent

In the assessment of the fines, it is also necessary to test its quality, understood as the presence of fine clays, which is measured with the sand equivalent test (*S.E*). According to the class of exposure to which the concrete will be subjected, the values should exceed 70% or 75%. None of the samples complied with the limits established in the standard due to the presence of mortars and ceramic elements, which behave as clays, as indicated in Appendix 15 of the EHE-08 [11]. Nevertheless, if the particle size of samples is adjusted for strict consideration as coarse aggregate (>4 mm), this restriction could be avoided.

#### 4.2.4. Density

Although the EHE-08 does not establish limits for any type of density—apparent (ρ_a_), after oven-drying (ρ_rd_) and saturated surface (ρ_ssd_), these are essential physical properties in the concrete mix design. The International Union of Laboratories and Experts in Construction Materials, Systems and Structures (RILEM) [27] recommends that if dry particles come from building rubble, they should have a minimum density of 1.5 Mg/m^3^. As can be seen in Table 2, all the samples presented densities which varied within the same ranges as those obtained for other ceramic materials from 1.6 Mg/m^3^ to 2.65 Mg/m^3^ in a study carried out by Sánchez de Juan and Alaejos [5], and exceeded the value recommended.

Interestingly, the density of recycled aggregates was significantly lower than that of the natural aggregates, which ranged between 2.4 Mg/m^3^ and 3 Mg/m^3^; this difference is explained by the greater porosity of recycled aggregate, due both to its ceramic nature and the presence of adhered mortar [28]. This will imply an increased need for water and cement, rendering it more difficult to achieve the required levels of strength and durability in concrete [4,7,29].

#### 4.2.5. Water Absorption

In contrast, water absorption is limited in the EHE-08 [11] is up to 7% for recycled coarse aggregate. The determination of water absorption is a good indicator of the quality of the recycled aggregates, since these kinds of aggregates have a high absorption capacity that can be detrimental to the workability of the concrete mix [6,9].

In view of the results obtained (Table 4), which are similar to those reported by Agrela *et al.* [9], Mas *et al.* [10] and Barbudo *et al.* [30], only two of the aggregates studied would be suitable as partial replacement of the natural coarse aggregate (CDW3 and CDW8) since the rest do not comply with standard specifications. A higher absorption coefficient was observed with the increase in the percentage of masonry, which agreed with the findings of Gomes and de Brito [31].

However, there is the possibility of using them if pre-treated by saturation with water before incorporating them into concrete mixes [9], which would involve using 5% more water according to Etxeberria *et al.* [32]. Moreover, if the recycled aggregates were judged according to the RILEM [27] recommendations of a 20% limit all the samples would comply.

#### 4.2.6. Shape

In terms of shape, recycle aggregates from the CDW possess a different external morphology to that of natural aggregates, presenting sharp edges, angular outlines, variable shapes and a more or less flat surface, which can be seen with the naked eye. This external appearance is due both to the initial form of the waste (*i.e.*, bricks, tiles…) and the process of obtaining the aggregate through use of a jaw crusher. Consequently, this type of secondary aggregate exhibits higher flakiness index than natural aggregates [33], which could lead to a reduction in the quality of the concrete in terms of lower strength and durability, since the need for a large quantity of water, cement, and sand, worsens workability [26].

The shape of the coarse aggregates was determined by calculating the flakiness index as the percentage by weight of flaky and needle-like aggregates is obtained, which should be less than 35% since higher values would make it difficult to achieve high strength concretes.

Table 4 shows the results obtained. The values obtained were in agreement with those reported by Mas *et al.* [10], except for one case, CDW11, the flakiness index values comply with the specifications given in the EHE-08 [11]. Given that there is a narrow margin for non-compliance, and that the non-compliance was due to the large quantity of ceramic waste in the form of bricks contained in the sample and to the plant treatment method, one possible solution may lie in employing a different crushing procedure, as it was observed that other samples with the next higher percentages of ceramic waste (CDW6 and CDW10) did not present this problem.

#### 4.2.7. Resistance to Fragmentation

The EHE-08 [11] recommends the use of the Los Angeles test to assess resistance to erosion of coarse aggregates through abrasion, wear, and impact, setting a value of 40% as the limit.

Although the Los Angeles test has been identified by several authors [34,35] as a constraint property in the use of CDW, since the presence of ceramic and mortar implies less resistance to fragmentation [8], the results obtained (Table 4), which are similar to those reported by Mas *et al.* [10] and Vegas *et al.* [36], show that only CDW1, CDW2, CDW7, and CDW10 exceeded the limit of 40%. However, since only CDW10 exceed the 50% limit, the EHE-08 permits the use of the rest of them in concrete with a resistance lower than 30 N/mm^2^ if there are experimental studies to support such use.

#### 4.2.8. Overall Performance

Although recycled concrete aggregates are commonly considered the best option for a partial replacement of the natural coarse aggregate in the concrete manufacture, a recent study by Silva *et al.* [37] revealed that MixRA may have similar or even better physical properties for use in concrete and proposed a classification system, based on the recycled aggregates composition, but mostly on their physical properties. Following the aforementioned the performance-based aggregate classification system, the overall performance of the recycled aggregates was assessed (Table 3).

**Table 3:** Performance-based classification of the recycled aggregates [37].

Sample	Class	Type
CDW1	C	I
CDW2	C	I
CDW3	B	II
CDW4	C	I
CDW5	B	III
CDW6	C	III
CDW7	C	II
CDW8	B	II
CDW10	D	-
CDW11	C	I
CDW12	C	I
CDW13	C	I

As expected, the majority of recycled aggregates characterized belong to Class C and only one sample was included in Class D. Therefore, according to Silva *et al.*’s [37] recommendations, these aggregates should only be considered for use in low-grade applications (*i.e.*, non-structural concrete). However, it is worth mentioning that as most of the aggregates failed the classification systems, hence, were included in a lower category, in terms of maximum water absorption (CDW2, CDW5, CDW6, CDW7, CDW12, and CDW13), an improvement could be achieved in their performance if some adjustments in the CDW treatment plant process were made. The results of a study [38] showed that, after the recycled aggregates were washed, the water absorption values fell by between 35% and 55% due to the removal of the finer particles. Consequently, the recycled aggregates obtained may be susceptible to be used in higher-level applications with the addition of a relatively simple procedure in the treatment of the CDW.

Additionally, three of the recycled aggregates studied (CDW3, CDW5 and CDW8), all of them previously classified as MixRA, belong to Class B, where most of the RCA could be placed, which indicate that some MixRA could present a high potential to be used in higher level applications, such as in partial replacement of the natural coarse aggregate in concrete manufacturing.

## 5. Conclusions

The use of recycled aggregates from construction and demolition in the concrete manufacture requires that the secondary materials possess the sufficient technical requirements in order to qualify as a material for potential use in construction. Thus, a quality assessment was done by the comparison between the test results obtained and the limits established in the Spanish legislation to ensure the compliance.

One of the problems with this approach is that recycled aggregates are characterized using traditional test methods, regardless of whether these are valid for the new materials. Consequently, establishing requirements for recycled aggregates on the basis of tests that may not be appropriate leaves these materials at a distinct disadvantage compared to conventional aggregates and creates a barrier to their use.

Even so, and albeit the use of MixRA or CerRA has yet to be allowed by Spanish legislation, the results obtained for the characterization demonstrate that the untreated recycled aggregate samples with different ceramic contents (MixRA and CerRA) shows promising values for their use in concrete manufacturing if some simple pretreatments are carried out to fix some quality issues. Nonetheless, it is necessary to emphasize that the results obtained are the product of an analysis of the recycled aggregates from CDW, collected at a specific day of sampling, in each of the CDW treatment facilities, thus, the reproducibility over time within the same management plant is not ensured; although similar results should be expected if recycled aggregates with a similar composition are tested.

In terms of composition, some of samples present a problem with the content of impurities, as the gypsum content reported could cause a non-compliance drawback with the requirements of soluble sulphates in acid that could be solved by carrying out a pretreatment of manual elimination of gypsum, which, as well, will remedy the problem of samples with a presence of impurities content over 1%.

The physical-mechanical characterization shows that the untreated recycled aggregates complied in terms of particle size distribution, particle size, density, shape, and resistance to fragmentation with the requirements stipulated in the EHE-08 for use as a coarse aggregate in the manufacture of concrete. For the rest of parameters which the recycled aggregates presented a deviation with regard to the regulatory limits, as it is the case for fines and water absorption, a solution could be found by carrying out a pre-treatment to adjust particle size and water saturation of the recycled aggregate. Finally, some non-fulfillments, *i.e.*, flakiness index and resistance to fragmentation, are impossible to alleviate by means of a pre-treatment and the recycled aggregate should be rejected for its use in concrete mixes but could be used in low level applications.

In conclusion, although the absence of specific legislation permitting its use, if controlled the production process in the CDW treatment plant and pretreated previous to its use in the concrete mix this type of recycled aggregate of ceramic origin has enough quality to become an input in the production of structural concrete.
